# RNA Interference of Four Genes in Adult *Bactrocera dorsalis* by Feeding Their dsRNAs

**DOI:** 10.1371/journal.pone.0017788

**Published:** 2011-03-18

**Authors:** Xiaoxue Li, Mingyan Zhang, Hongyu Zhang

**Affiliations:** State Key Laboratory of Agricultural Microbiology, Hubei Key Laboratory of Insect Resource Application and Sustainable Pest Control and Institute of Urban and Horticultural Pests, College of Plant Science and Technology, Huazhong Agricultural University, Wuhan, Hubei, People's Republic of China; New Mexico State University, United States of America

## Abstract

**Background:**

RNA interference (RNAi) is a powerful method to inhibit gene expression in a sequence specific manner. Recently silencing the target gene through feeding has been successfully carried out in many insect species.

**Methodology/Principal Findings:**

*Escherichia coli* strain HT115 was genetically engineered to express dsRNA targeting genes that encode ribosomal protein Rpl19, V type ATPase D subunit, the fatty acid elongase Noa and a small GTPase Rab11. qRT-PCR showed that mRNA level of four target genes was reduced compared to ds-*egfp* control by feeding either engineered bacteria or dsRNAs. The maximum down-regulation of each gene varied from 35% to 100%. Tissue specific examination indicated that RNAi could be observed not only in midgut but also in other tissues like the ovary, nervous system and fat body. Silencing of *rab11* through ingestion of dsRNA killed 20% of adult flies. Egg production was affected through feeding ds-*noa* and ds-*rab11* compared to ds-*egfp* group. Adult flies were continuously fed with dsRNA and bacteria expressing dsRNA for 14 days and up-regulations of target genes were observed during this process. The transcripts of *noa* showed up-regulation compared to ds-*egfp* control group in four tissues on day 7 after continuous feeding either dsRNA or engineered bacteria. The maximum over-expression is 21 times compared to ds-*egfp* control group. Up-regulation of *rab11* mRNA level could be observed in testes on day 7 after continuous bacteria treatment and in midgut on day 2 after ds-*rab11* treatment. This phenomenon could also be observed in *rpl19* groups.

**Conclusions:**

Our results suggested that it is feasible to silence genes by feeding dsRNA and bacteria expressing dsRNA in *Bactrocera dorsalis*. Additionally the over-expression of the target gene after continuously feeding dsRNA or bacteria was observed.

## Introduction

The oriental fruit fly, *Bactrocera dorsalis* (Hendel), is a major pest throughout South East Asia and in a number of Pacific Islands, feeding on a wide variety of fruit crops such as citrus and guava [1].

RNA interference (RNAi), which is initially discovered in nematode *Caenorhabditis elegans*, is widely accepted as a powerful tool for gene function research [2]. In *C.elegans*, RNAi effects can be produced by feeding bacteria expressing dsRNA [3], [4], or even by soaking nematodes in dsRNA solution [5]. It is now clear that dsRNA-mediated gene silencing is a conserved mechanism in many eukaryotes [6], [7]. This post-transcriptional gene silencing technique has been used in entomological research [8]–[10]. Microinjection is used in most of these experiments to achieve high titer of dsRNA and expose more tissues to silencing factor.

Recent researchers have shown some successful examples indicating that dsRNA feeding could be used as a potential way for pest control. Baum et al. screened *Diabrotica virgifera virgifera* cDNA library to identify 290 putative target genes predicted to encode an essential protein. 125 dsRNA tested showed significant larva mortality and/or stunting at 52 ng/cm^2^ [11]. Other examples of RNAi through ingestion such as *Epiphyas postvittana* [12], *Reticulitermes flavipes* [13], *Plutella xylostella* [14], *Rhodnius prolixus* [15], *Spodoptera frugiperda* [16] have also been proven to be a feasible way for agricultural pests control. Because high concentration of dsRNA is necessary to ensure continuous suppression of target genes [17], these experiments which are based on the methods of feeding *in vitro* synthesized dsRNA to the larva are relatively expensive. Using genetically engineered *Escherichia coli* strain HT115 to express dsRNA is an economical way to produce large quantities of dsRNA. Besides, bacterial feeding is a non-disrupting technique preserving the integrity of the treated animals. *E.coli*-mediated delivery of dsRNA has been reported in *C. elegans* [4], planarians [18], *Entamoeba histolytica* [19] and *Spodoptera exigua* [17].

However, in *Drosophila melanogaster*, feeding yeast cells engineered to express double-stranded RNA to the flies failed to work [20]. Besides, RNAi-mediated gene knockdown through microinjection in *Drosophila* is localized to the site of dsRNA delivery and effects are temporally limited [21]. Furthermore, injection of dsRNA into haemolymph or gut didn't trigger systemic gene silence [22]. These facts make people believe that in Diptera species, feeding dsRNA cannot induce RNAi. However, in *B.dorsalis*, microinjection of dsRNA into adult abdomen successfully inhibits the expression of *doublesex* gene in ovary [23]. Thus, although *B.dorsalis* belongs to Diptera, feeding dsRNA may work in *B.dorsalis*.

The *sid-1* (systemic RNA interference deficient-1) gene is essential and sufficient to mediate systemic RNAi effect in *C.elegans* [24]. Up till now, *sid-1* genes have been found in almost all animal genomes with the exception of Dipteran genomes; this may be one reason why feeding *D.melanogaster* yeast-expressed dsRNA failed [21]. Some evidences suggested that uptake of dsRNA in S2 cells involves a receptor-mediated endocytosis [25]. Endocytosis of dsRNA also seems to occur in *C.elegans* because knockdown of components of the endocytotic pathway by RNAi results in worms with a ‘loss of RNAi function’ phenotype [25]. These results suggest that receptor-mediated endocytosis is a widespread mechanism for dsRNA uptake [21]. Based on the known information, we design the experiments to test whether dsRNA feeding and systemic RNAi exist in *B.dorsalis*.

Since killing adult flies or cutting back the number of eggs could both reduce the damage to the fruits, four genes were chosen to test the feasibility of RNAi in *B.dorsalis*. *rpl19* and *v-ATPase-D* are housekeeping genes and are very conservative both in structure and in function. Loss of the functions could cause death in flies. In the dsRNA feeding bioassay on west corn rootworm, both *rpl19* and *v-ATPase-D* caused high mortality in larva, the LC_50_ are 5.20 ng/cm^2^ and 1.72 ng/cm^2^ [11]. *rpl19* encodes a ribosomal protein L19 which is a component of the 60S subunit belonged to the L19E family of ribosomal proteins [26]. *v-ATPase-D* encodes the subunit D of the V_1_ domain of V-ATPase, which forms a central rotor axle together with subunit F [27]. *noa* and *rab11* are chosen as the target in order to reduce egg production. *noa* encodes a very long chain fatty acid elongase, which exists in the nervous system, the imaginal discs, the fat body and in the gonads of both sexes [28]. An RNAi construct of *noa* leads to male sterility in *D.melanogaster*, demonstrating the necessity of *noa* function for male germline development [28]. Rab11, which is a small GTPase that regulates several aspects of vesicular trafficking, is essential for fertility in *D.melanogaster* and plays a key role in regulating various cellular events during *D.melanogaster* development and differentiation [29], [30].

Here we explored the feasibility of feeding dsRNA to adult *B. dorsalis* to achieve RNAi. The results of qRT-PCR indicated that directly feeding dsRNA and feeding dsRNA-expressing bacteria both induced RNAi in *B.dorsalis*. Furthermore, continuous feeding dsRNA led to over-expression of target genes. The death of adult flies could be observed in the *rab11* dsRNA bioassay. In the bioassay targeting *noa* and *rab11*, egg production are reduced.

## Results

### Cloning of partial CDS of *noa*, *rab11*, *rpl19* and *v-ATPase-D*

RT-PCR was used to amplify the partial sequence of the four target genes. The partial CDS we obtained for the four genes covered approximately 51% of *noa*, 92% of *rab11*, 66% of *rpl19*, 54% of *v-ATPase-D* full CDS respectively (GenBank accession number HQ148712, HQ315779, HQ315780 and HQ315781). The Blastn results showed high identity to *D.melanogaster*, each about 83%, 82%, 76% and 79% for *noa*, *rab11*, *rpl19* and *v-ATPase-D*.

### Stability of dsRNA and confirmation of ingestion of bacteria in the midgut

**Figure 1:**
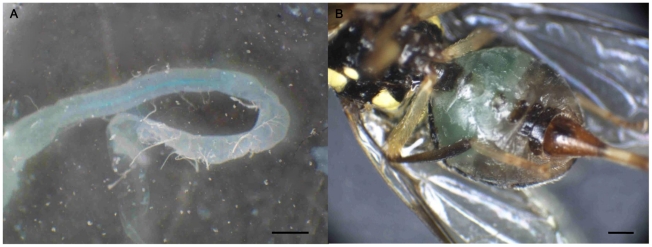
Ingestion and accumulation of bacteria in the midgut of *Bactrocera dorsalis*. (A) The midgut from the adult fed on FD&C blue colored bacteria for 3 days. (B) Blue color accumulated in the abdomen of adult fed by FD&C blue for 3 days. Scale bars: 0.5 mm.

**Figure 2:**
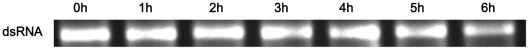
dsRNA stability on artificial diet. dsRNA on artificial was extracted and examined by electrophoresis for integrity every one hour.

The midgut dissected from the flies fed by FD&C colored bacteria showed the blue color clearly (Figure 1A). The bacteria turned the abdomen of the fly to blue (Figure 1B). This experiment demonstrated that the bacteria accumulated in the midgut of the flies through ingestion. The results of electrophoresis demonstrated that the dsRNA exists 6 h after being added on the surface of the artificial diet (Figure 2). Considering that the feeding time of *B.dorsalis* is 8:00 to 10:30 and 15:00 to 18:00, replacing the artificial diet with new dsRNA or bacteria twice a day at 8:00 and 14:00 would be enough to ensure the validity of the dsRNA feeding methods [31].

### Ingestion of dsRNA or dsRNA-expressing bacteria induced RNAi in *Bactrocera dorsalis*

**Figure 3:**
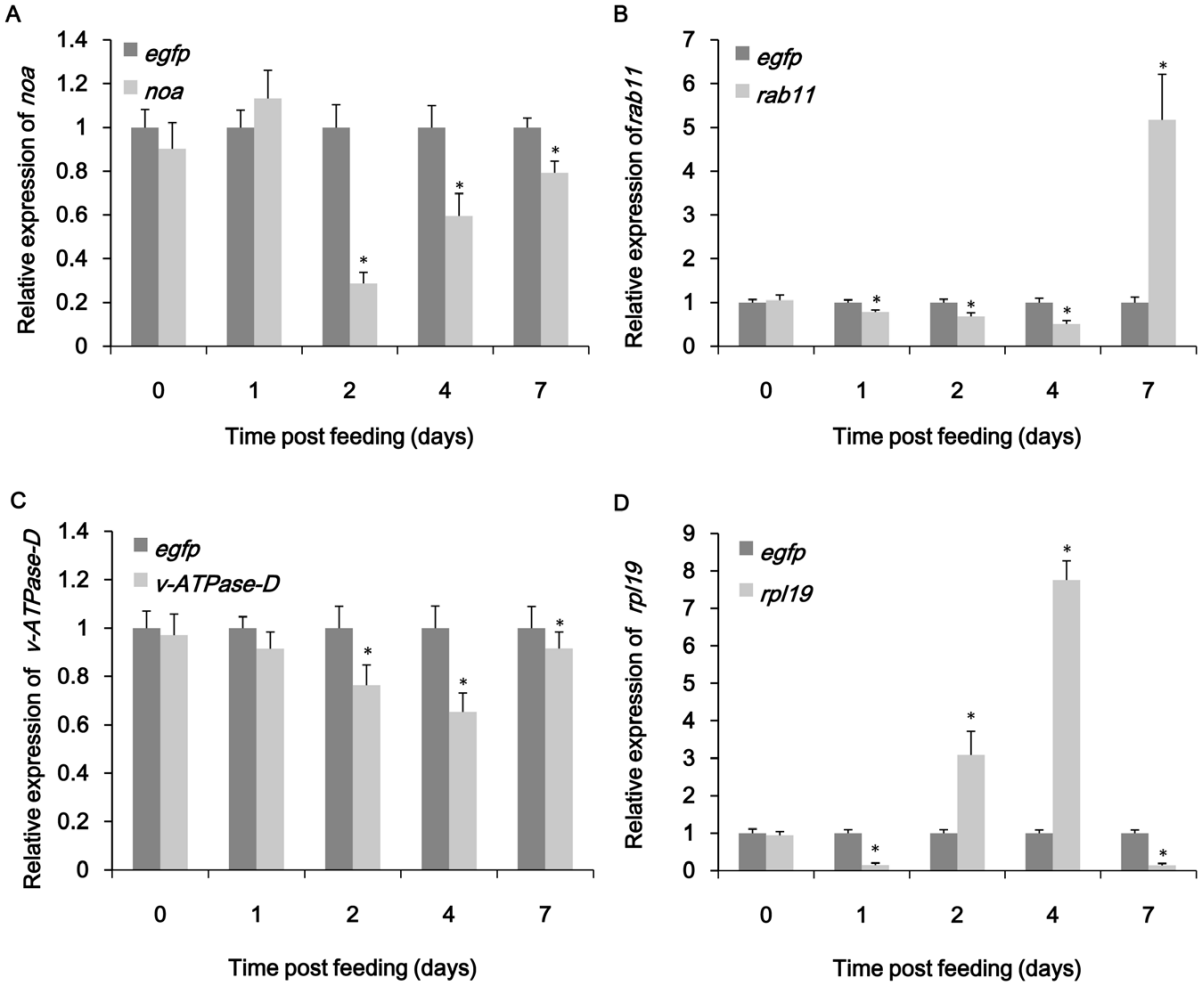
Knockdown of *noa* (A), *rab11* (B), *v-ATPase-D* (C) and *rpl19* (D) expression by ingestion of dsRNA. The samples of each group were collected on day0, day1, day2, day4 and day7. ds-*egfp* was used as control. All the experiments were triplicated. Asterisk stands for significant difference of relative expression. (p<0.05, Duncan's test).

**Figure 4:**
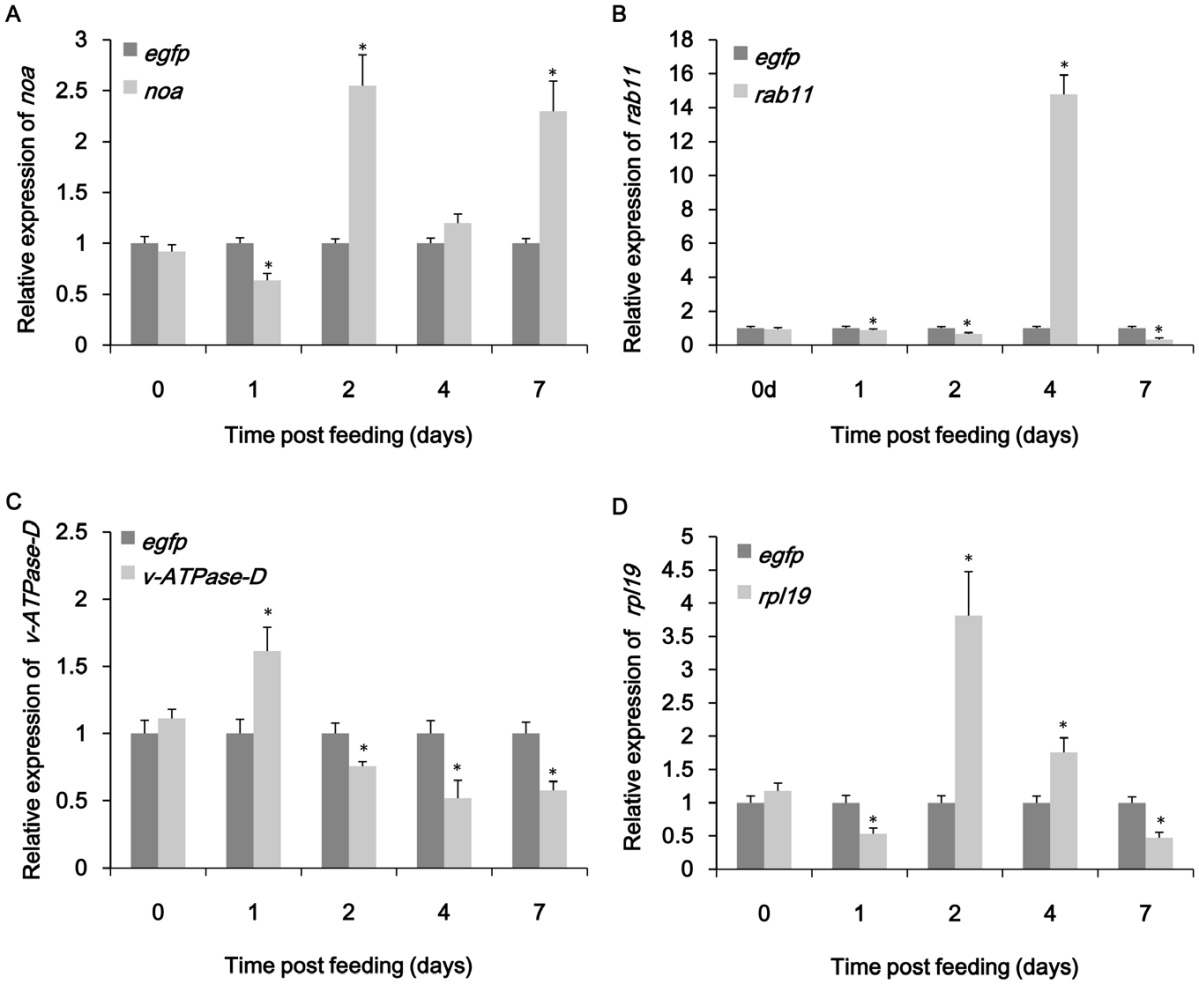
Knockdown of *noa* (A), *rab11* (B), *v-ATPase-D* (C) and *rpl19* (D) expression by ingestion of bacteria expressing dsRNA. The samples of each group were collected on day0, day1, day2, day4 and day7. Bacteria expressing ds-*egfp* was used as control. All the experiments were triplicated. Asterisk stands for significant difference of relative expression. (p<0.05, Duncan's test).

To confirm that ingestion of dsRNA triggered a specific RNAi in *B.dorsalis* adult flies, qRT-PCR was performed to detect the mRNA level of each target genes. The experiment was designed following the MIQE instructions [32]. The results showed that ingestion of dsRNA or dsRNA-expressing bacteria both induced RNAi in *B.dorsalis*. Despite a high variability between different groups, something common could be found in the results. Among four groups directly fed with dsRNA solution, all four genes showed significant drop of target transcript compared to control group on certain day respectively (Fig. 3A, B, C and D). The maximum reduction of each target gene varied from 50% to 82%. The lowest point of *noa* transcript occurred on day 2 with a 71% decrease compared to ds-*egfp* control(Fig. 3A). The *rpl19* mRNA showed obvious down-regulation on both day 1 and day 7 with a 85% decrease compared to control group (Fig. 3D). ds-*rab11* and ds-*v-ATPase-D* treated group showed maximum down-regulation both on day 4 (Fig. 3B and D). The situation was somewhat different in the experiment of ingestion of bacteria expressing dsRNA. The reductions of target genes were not as significant as dsRNA feeding group. For example, only a 36% decrease of *noa* transcript and a 53% decrease of *rpl19* transcript were observed during the bioassay (Fig. 4A and D). The effect of bacteria expressing ds-*rab11* was not as significant as in dsRNA feeding group (Fig. 4B). Although suppression of targeted mRNAs could be observed by qRT-PCR, gross effects on morphology, growth and development were not apparent.

### Continuously feeding dsRNA induced over-expression of the target genes

Interestingly, the expression of the four genes exhibited up-regulation notwithstanding the continuous feeding dsRNA-expressing bacteria or dsRNA for 14 days. Both bacteria and dsRNA treatment of *rpl19* and *rab11* induced over-expression of their mRNA levels. The peak of ds-*rpl19* treated group appeared on day 4 with 7.8 times as high as ds-*egfp* control (Figure 3D) while in ds-*rpl19* bacteria treated group the maximum expression appeared on day 2 (Figure 4D). *rab11* showed 14.8 times up-regulation on day 4 in bacteria group (Figure 4B) and 5.2 times up-regulation on day 7 in ds-*rab11* group (Figure 3B). For *noa*, the situation was different between dsRNA and bacteria treatment. Continuous feeding ds-*noa* directly did not cause significant over-expression (Figure 3A). However, in bacteria treatment, over-expression occurred on day 2 and day 7 (Figure 4A). Two tests on *v-ATPase-D* did not induce obvious over-expression compared to other genes with only first day showed 1.6 times up-regulation in bacterial treatment (Figure 4C).

### Confirmation of gene knockdown in specific tissues

**Figure 5:**
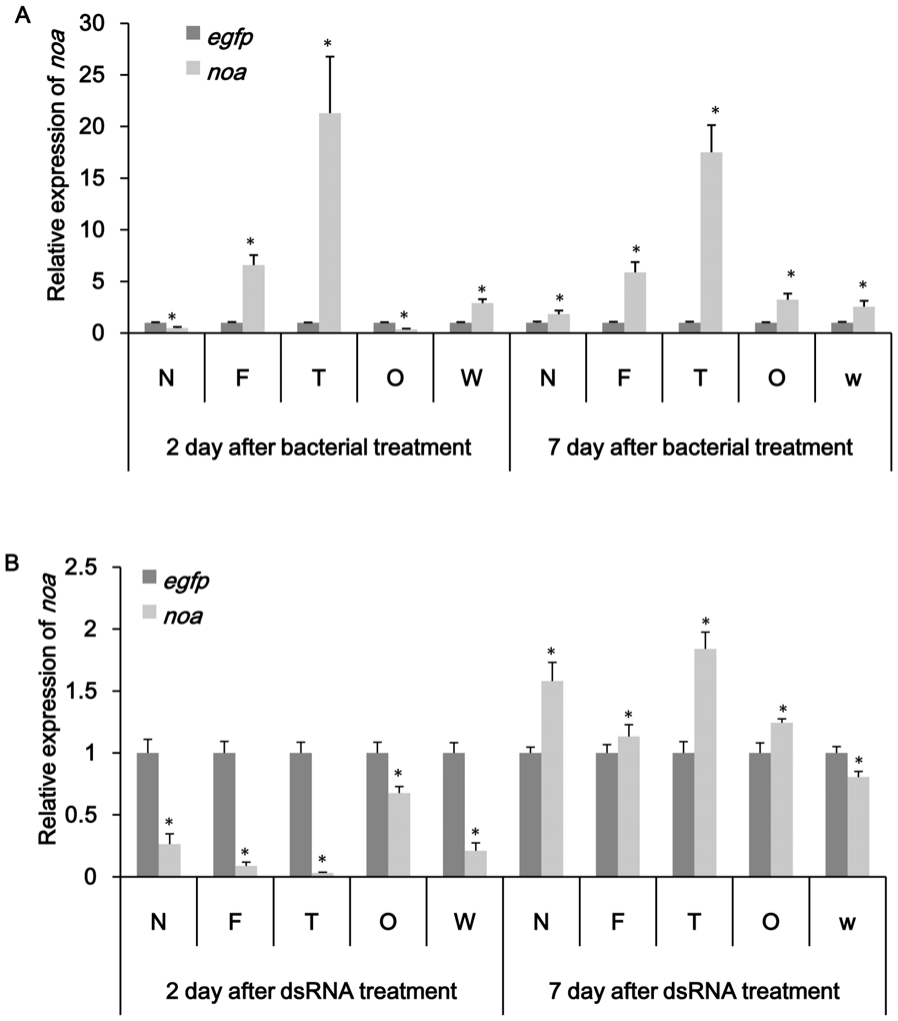
Relative expression of *noa* in specific tissues. (A) Expression of *noa* after feeding bacteria expressing ds-*noa*. (B) Expression of *noa* after feeding ds-*noa*. N: nervous system; F: fatbody; T: testes; O: ovary; W: whole insect. *egfp* was used as control. All the experiments were triplicated. Asterisk stands for significant difference of relative expression. (p<0.05, Duncan's test).

**Figure 6:**
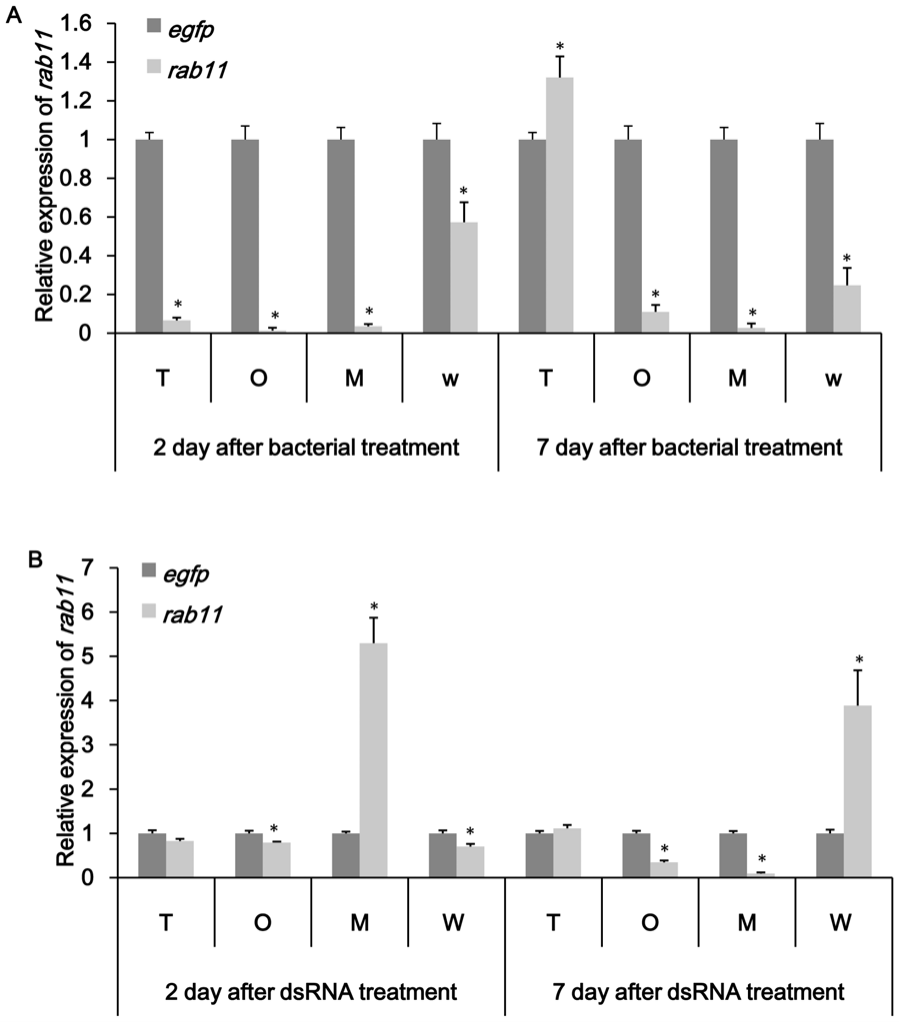
Relative expression of *rab11* in specific tissues. (A) Expression of *rab11* after feeding bacteria expressing ds-*rab11*. (B) Expression of *rab11* after feeding ds-*rab11*. M: midgut; T: testes; O: ovary; W: whole insect. *egfp* was used as control. All the experiments were triplicated. Asterisk stands for significant difference of relative expression. (p<0.05, Duncan's test).

**Figure 7:**
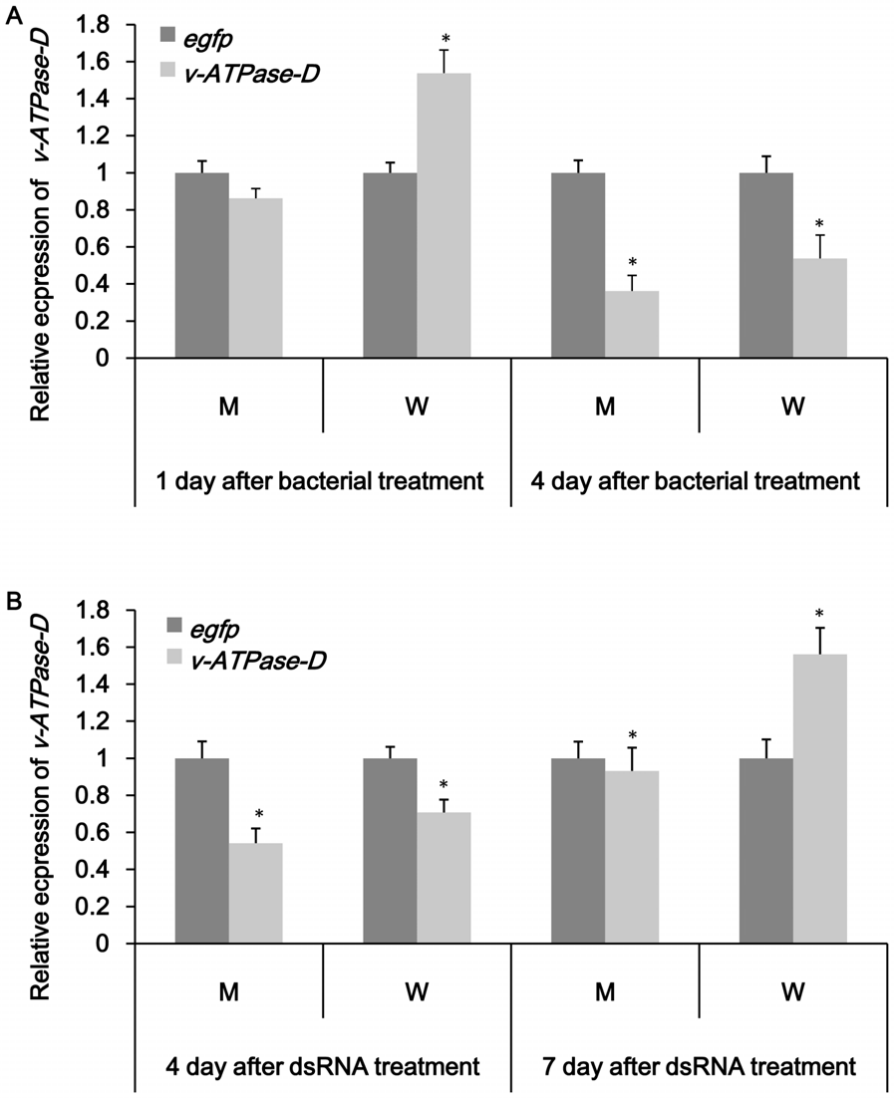
Relative expression of *v-ATPase-D* in specific tissues. (A) Expression of *v-ATPase-D* after feeding bacteria expressing ds-*v-ATPase-D*. (B) Expression of *v-ATPase-D* after feeding ds-*v-ATPase-D*. M: midgut; W: whole insect. *egfp* was used as control. All the experiments were triplicated. Asterisk stands for significant difference of relative expression. (p<0.05, Duncan's test).

**Figure 8:**
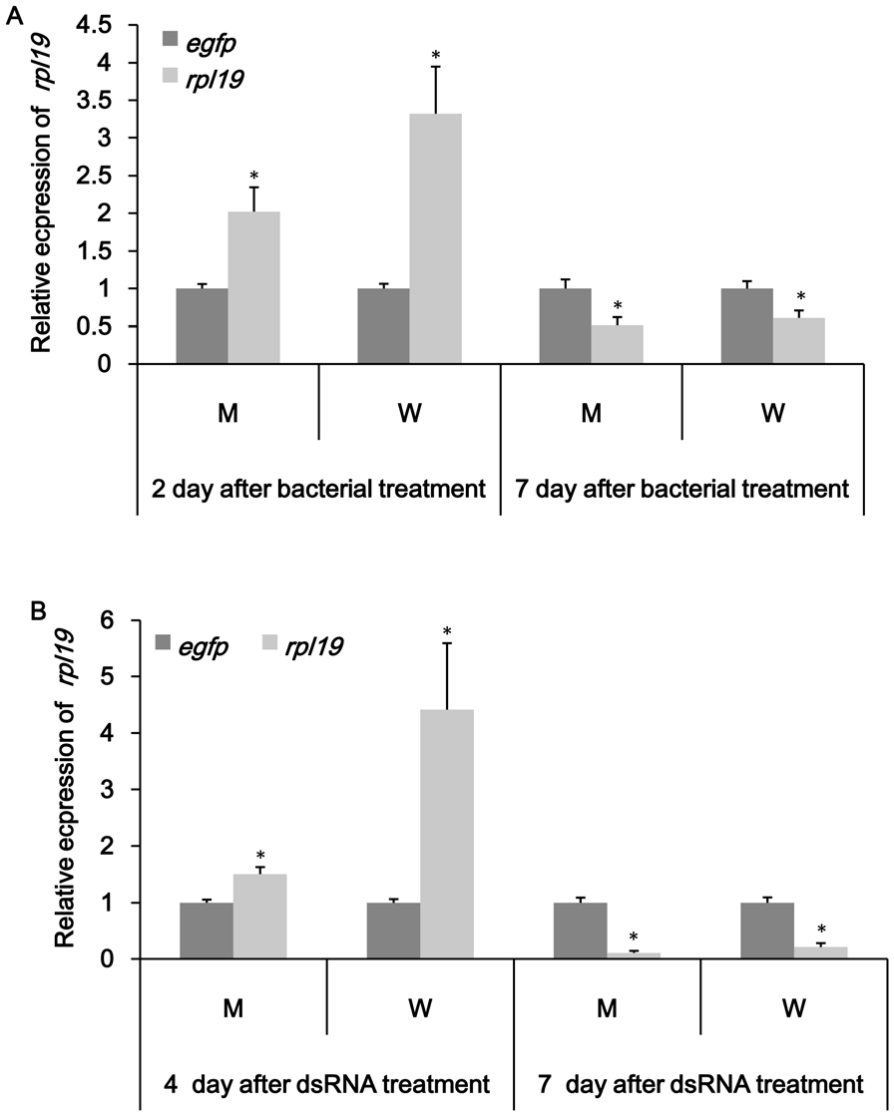
Relative expression of *rpl19* in specific tissues. (A) Expression of *rpl19* after feeding bacteria expressing ds-*rpl19*. (B) Expression of *rpl19* after feeding ds-*rpl19*. M: midgut; W: whole insect. *egfp* was used as control. All the experiments were triplicated. Asterisk stands for significant difference of relative expression. (p<0.05, Duncan's test).

The results showed that both feeding dsRNA and engineered bacteria reduced the expression of target genes in specific tissues. *noa* transcripts in nervous system, fat body, ovary and testes were significantly affected in both treatment (Figure 5A and B). It was also easy to see that RNAi effect was more obvious in tissues of interest compared to whole insect. All tissues exhibited a decrease of *noa* expression on day 2 and up-regulation on day 7 in dsRNA treated group. Bacteria expressing ds-*noa* induced higher over-expression than directly feeding ds-*noa*. In bacteria treated group, fatbody and testes showed up-regulation both on day 2 and day 7. *rab11* transcripts in target tissues such as midgut, ovary and testes were obviously reduced in bacteria treatment groups except *rab11* mRNA level in testes were up-regulated (Figure 6A). Compared to bacteria treatment, obvious silence could not be observed till day 7 in midgut and ovary in ds-*rab* treatment group (Figure 6B). For *v-ATPase-D* and *rpl19* group, midgut was dissected to confirm the tissue-specific knockdown. *v-ATPase-D* expression in midgut was quite similar with the results of whole insect and previous results. Both up-regulation and down-regulation were not obvious using either method (Figure 7A and B). *rpl19* mRNA level in midgut was almost the same as in whole insects in both treatments (Figure 8A and B).

### Ingestion of certain dsRNA caused death in adult flies and influenced egg production

**Figure 9:**
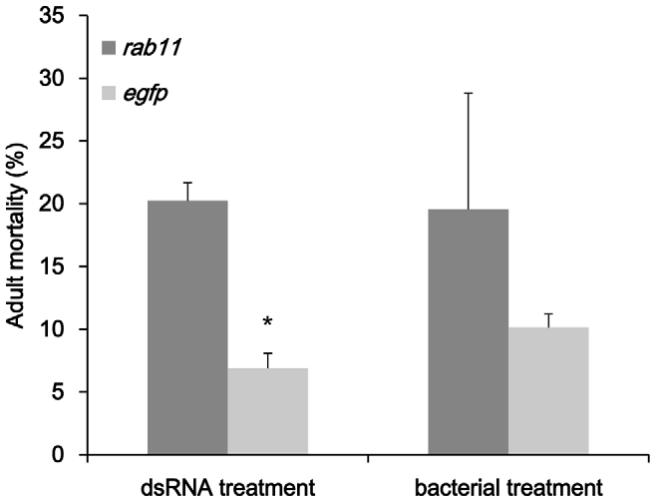
Mortality rate of *rab11*. Mortality rate of adult flies in dsRNA treatment and bacteria treatment in 14 days bioassay was caculated. Asterisk stands for significant difference of relative expression. (p<0.05, Duncan's test).

**Figure 10:**
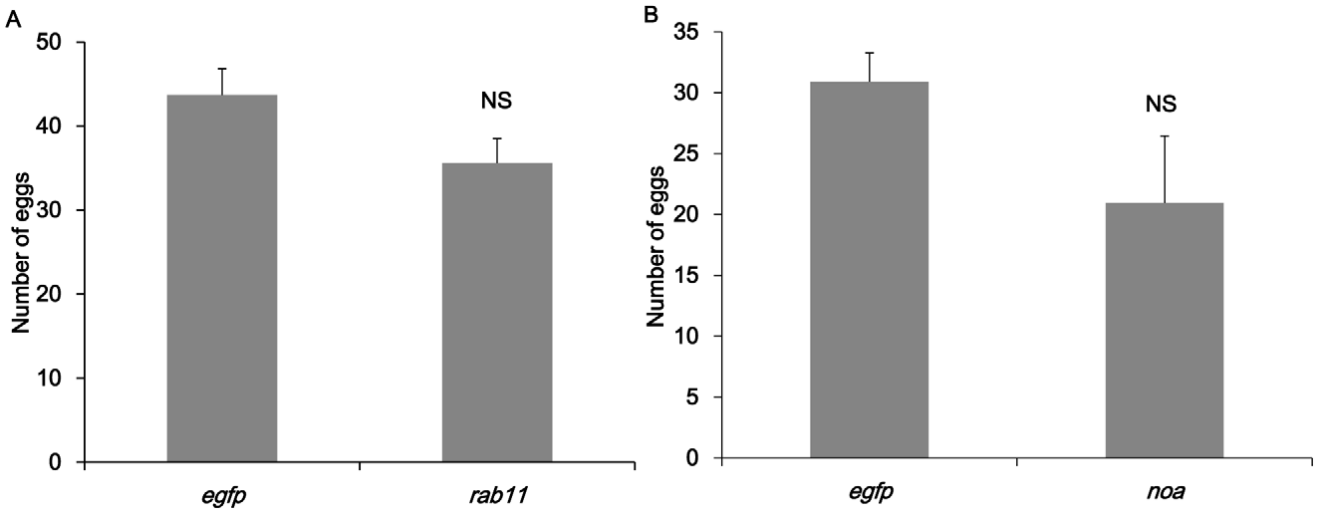
Reduction of egg-laying resulting from RNAi effects. (A) number of eggs laid by ds-*rab11* treated females and males (B) number of eggs laid by normal virgin females mating with ds-*noa* treated males. ds-*egfp* treatment group was used as control. NS stands for that there was no significant difference at p<0.05 level.

Our results were different from the data of Baum's group which relative low dose of *rpl19* and *v-ATPase-D* dsRNA caused high mortality to the larva WCR. In the bioassay done on *B.dorsalis*, only *rab11* caused relatively high death rate (Figure 9). The largest death rate occurred in the group feeding on ds-rab*11*. Approximately 21% flies died in the bioassay which is significantly higher than control group (p<0.05). *rpl19*, *v-ATPase-D* and *noa* dsRNA didn't cause significant death in *B.dorsalis* adult flies (data not shown). The eggs laid by ds-*noa* and ds-*rab11* treated females were also collected and examined under microscope (Figure 10). In *ds-noa* treated group, each female could produce approximately 21 eggs per day in average which is a 32% drop compared to the control. A 20% drop of egg production was observed in the females from ds-*rab11* treated group. However, no morphological changes were observed in eggs or reproductive systems.

## Discussion

In this study we demonstrated that ingestion of dsRNA and bacteria expressing dsRNA can inhibit the transcription of target genes in *B.dorsalis*. Furthermore, this effect is not limited to the genes which are expressed in midgut. Before the bioassay, we try to obtain the *sid-1* gene which is crucial for the systemic RNAi effects. However, using degenerated primers designed based on the conserved domain of *sid-1*, we didn't get the expected fragment. Although *B.dorsalis* lacks *sid-1* gene, the success of RNAi in *B.dorsalis* suggest that other factors might explain this phenomenon. In fact, previous research confirms this result. Saleh et al. demonstrates that dsRNA uptake into cultured *Drosophila* S2 cell involving a receptor-mediated endocytosis rather than a sid-1 mechanism. Moreover, endocytosis of dsRNA also seems to occur in *C.elegans* [25]. Their recent study demonstrated that infected cells spread systemically silencing signal after virus infection throughout *D.melanogaster* which requires endocytosis [33]. These results suggest that uptake of dsRNA through endocytosis might also occur in *B.dorsalis* instead of *sid-1* based mechanism.

Feeding dsRNA or engineered bacteria both induced the silence of target genes. dsRNA ingested through diet caused significant drop of transcript level in *B.dorsalis* compared to control group. A maximum 71% decrease was observed on day 2 for *noa* group, 50% decrease for *rab11* on day 4, 35% decrease for *v-ATpase-D* on day 4 and 86% decrease for *rpl19* on day 7 through ingestion of each dsRNA. The RNAi effect of feeding bacteria expressing dsRNA was not as obvious as feeding dsRNA. For example, only 36% drop could be observed on day 2 in bacteria expressing ds-*noa* group. It was same with the other 3 genes. Our results were consistent with previous research using oral delivery of dsRNA method. Feeding dsRNA to the termite *R. flavipes* reduced *Cell-1* and *Hex-2* mRNA relative expression by nearly 60% on day 2 [13]. Salivary gland nitrophorin 2 demonstrated a significant decrease of 42% 48 h after ingestion dsRNA in *R. prolixus* [15]. Other successful examples in *S. frugiperda* and *E. postvittana* also indicated that feeding dsRNA could silence target gene expression in many insect orders [12], [16]. qRT-PCR results suggested that feeding dsRNA to *B.dorsalis* could achieve the same silence effect on mRNA level as in other insects.

In this experiment, tissue specific RNAi effect could be observed in midgut, nervous system and reproductive system. For *noa* and *rab11*, RNAi effect was more obvious in target tissues like ovary and testes compared to whole insect. For example, *noa* transcript level exhibited 93% and 87% decrease in testes and ovary respectively 2 days after bacteria treatment compared to 43% in whole insect. It was the same about the expression of *v-ATpase-D* and *rpl19* in midgut and whole insect after bacteria and dsRNA treatment. Previous studies also indicated that RNAi effect were not limited to genes expressing in midgut. Using *in vitro* synthesized dsRNA feed *E. postvittana* larva successfully silences the expression of pheromone binding protein gene in antenna [12]. Ingestion of dsRNA can effectively inhibit gene expression in *S. exigua* when insects are fed with dsRNA targeting non-midgut genes [17]. These facts suggested that delivery of dsRNA through ingestion could inhibit the gene expression not only in midgut but also in other parts of insect. Considering that midgut and associated structures are the only regions uncovered by chitin exoskeleton, uptake of dsRNA probably happens in midgut [21]. Thus, how the effect transferred from midgut to other part of the insect need to be further discussed.

Based on the effect of dsRNA on transcript levels of target genes, we would expect a high mortality happened in *v-ATpase-D* and *rpl19* feeding research and reduced egg production in *rab11* and *noa*. Furthermore, *v-ATPase-A* and *rpl19* also caused high mortality in the test of WCR feeding assays at relatively low dose [11]. Price and Gatehouse have also mentioned that the demonstrated efficacy of targeting *v- ATPase-A* could easily be extended to other insect species [21]. Besides, in *D.melanogaster*, the *rab11* mutant males and females are sterile [30]. However, in our bioassay, the mortality of both *rpl19* and *v-ATpase-D* are relatively low (data not shown) in spite of high dose of dsRNA compared with the LC_50_ for the same genes in the research of Baum et al. on WCR larva [11]. Ingestion of ds-*rab11* and ds-*noa* did not reduce the egg production significantly. Several reasons might contribute to this phenomenon. First, in this experiment, adult flies were chosen as the object of the research. Quite a few studies were focused on adult insect using feeding method. In the research by Baum et al., larva was used as the target for WCR assay [11]. Moreover, it is highly possible that different RNAi mechanism exists in Diptera species as described above.

Over-expression of target genes was observed in *B.dorsalis* for the first time. It is not clear about the mechanisms involved. This phenomenon may due to either stress response triggered by RNAi or resistance to RNAi which might exists in insect. Some previous study may offer some inspiration to us. First, mammalian hosts have involved with some defense mechanism against dsRNA virus. The infection triggers a signal cascade activating interferon stimulated gene (ISGs) [34]. Recent studies indicate that siRNA and shRNA targeting particular gene can also induce nonspecific effects in cells by activating ISGs involved in the stress response pathways [35], [36]. The level of activation observed varies depending on the concentration of the RNAi molecule or on cell line. Some studies have also indicated that 3 signaling pathway are activated in antiviral process of *D.melanogaster*, Toll-way, IMD-way and Jak-STAT pathway [37], [38]. Actually, transfection of siRNAs results in interferon (IFN)-mediated activation of the Jak–STAT pathway and global upregulation of IFN-stimulated genes in human cells [35].These facts suggest that stress response to RNAi may play a key role in the over-expression of target genes. Second, some evidences indicate it has been known for a while that some plant, insect and even some mammalian viruses have to counteract RNAi to invade their hosts [39]. This mechanism involved with mutation in the target regions, viral proteins as the suppressors of RNAi or the resistant to Dicer activity [40]–[44]. Although to date, no evidences have shown that either of these mechanisms exists in eukaryotes, these facts still offer a perspective of further study on the over-expression of target genes happened in RNA interference.

## Materials and Methods

### Insects

A laboratory strain of *B. dorsalis* was reared as experimental material. Adult flies were maintained in cages at 28°C under a 12 h light∶12 h dark photoperiod on artificial diets consisting of 2.5% yeast extract, 7.5% sugar, 2.5% honey, 0.5% agar and 87% H_2_O. Hatched larvae were cultured by periodic transfer to bananas.

### Cloning of target genes

Total RNAs from adult flies were extracted with RNAiso™ Plus (TaKaRa). First strand cDNA was produced from 1 µg RNA using PrimeScript™ 1st Strand cDNA Synthesis Kit (TaKaRa). Degenerated primers were designed using icodehop program (Table 1). PCR fragments were purified using Universal DNA purification Kit (TIANGEN), then cloned into pEASY-T1 Simple Cloning Vector (TRANSGEN) and sequenced.

**Table 1:** PCR primers used for RT-PCR and qRT-PCR analyses.

Primer	Sequence (from 5′ to3′)
D*rab11*-L	GCGATGATGAGTACGATTACCTGTTYAARGTNGT
D*rab11*-R	TGGGCTTCACATCGATGGSYTCNACRTT
D*noa*-L	CACTACGGCCTGCACCACWSNGTNTGYGT
D*noa*-R	GCGAACAGCACGAAGTAGGAGVWRTACATNGC
D*rpl19*-L	TGCGGTGCGGCAARAARAARGT
D*rpl19*-R	CTCGGCCTTCTTCTTGTGDATRTRYTC
D*v-ATpase d*-L	TCTGGGCTCCTTCGARCARATGARG
D*v-ATpase d*-R	TCAGGTAGGCGTAGAACACNCCRWARTG
S*rab11*-L	CGAGCTCTTGCTATCCCGCTTTACG
S*rab11*-R	CCCAAGCTTAAGAGTCTCGCCTCATCC
S*noa*-L	CGAGCTCCTGGACCTGGTTGTTTGTG
S*noa*-R	CCCAAGCTTATGAAGCGTGGCGGATTG
S*rpl19*-L	CGAGCTC GTTTGGCTGGACCCTAATGA
S*rpl19*-R	CCCAAGCTT GCATTCGAGCATTTGCAGTA
S*v-ATPase-D*-L	CGAGCTC CTGCAAGAAATTGGGTGGTT
S*v-ATPase-D*-R	CCCAAGCTT TGTCTTATCCCCAGGATTGG
S*egfp-L*	CGAGCTC ACGTAAACGGCCACAAGTTC
S*egfp-R*	CCCAAGCTT AAGTCGTGCTGCTTCATGTG
Q*rab11*-L	AAACATCTGCCCTTGATTCG
Q*rab11*-R	CTTCACATCGATGGGTTCAA
Q*noa*-L	GCAATTGACGCAAATGATTG
Q*noa*-R	GGCGATCGATAGGTTGATGT
Q*rpl19*-L	CGTCAACGTGTATTGAGACG
Q*rpl19*-R	GGCCTTCTTCTTGTGGATGT
Q*v-ATPase-D*-L	AGCAAATGGAGGCAATTCAT
Q*v-ATPase-D*-R	AAGAATGGTGCCAATGGTGT

Primers starting with a D were degenerated primers to get the partial CDS of each gene. Primers starting with an S were used to get PCR fragment for L4440 plasmid construction. Primers starting with a Q were designed for qRT-PCR. The underlined part indicates enzyme restrict site.

### Plasmid Construction and Expression of dsRNA

According to Baum's research, dsRNA prepared from different sections of coding region showed activity comparable to full length dsRNA [11]. So randomly chosen section on coding region of each target gene was amplified from adult flies' cDNA. In order to construct the plasmid expressing dsRNA, fragments of each four genes were amplified by RT-PCR using specific primers (Table 1). *egfp* fragment which was used as control was amplified from Pub·nls·EGFP (Provided by Dr. Handler, USDA). The underlined portions of sequence were SacI and HindIII restriction sites respectively. PCR products were then cloned into MCS of L4440 plasmid. The L4440 plasmid, which was obtained from Genecool, has two T7 promoters in inverted orientation flanking the multiple cloning site. The plasmid constructs L4440-*rpl19*, L4440-*rab11*, L4440-*v-typeATPase-D*, L4440-*noa* were verified by PCR, restriction analysis and sequencing.

HT115(DE3) competent cells lacking RNase III were prepared using standard CaCl_2_ methodology and were transformed with recombinant plasmid DNA. Single colonies of HT115(DE3) were cultured in LB at 37°C with shaking at 220 rpm overnight. The culture was diluted 100-fold in 800 ml 2×YT supplemented with 75 µg/ml ampicillin plus 12.5 µg/ml tetracycline and cultured at 37°C to OD600 = 0.5. Synthesis of T7 polymerase was induced with 0.4 mM IPTG and the bacteria were incubated with shaking for an additional 4 h at 37°C. For bacteria feeding experiments, bacteria were centrifuged at 5,000 g for 10 min and resuspended in ddH_2_O to 250×.

### Quantification and Purification of dsRNA

Total nucleic acids were extracted as described by Fire et al [4]. Bacteria cultures were centrifuged at 5,000 g for 10 min. Cell pellets were resuspended in 1 M ammonium acetate/10 mM EDTA plus the same volume of phenol∶chloroform∶isoamyl alcohol (25∶24∶1). The samples were incubated at 65°C for 30 min and centrifuged at 12,000 g for 15 min. The upper phase was mixed with isopropanol, incubated at −20°C overnight and centrifuged at 12,000 g for 30 min. The nucleic acid was treated with RQ1 RNase free DNase (Promega) to remove single strand DNA and RNase A solution (Promega) to remove single strand RNA. The nucleic acid pellets were resuspended in 1×TE buffer, pH 7.5 and loaded onto a 2% agarose TBE gel, stained with ethidium bromide, and photographed. The concentration was determined by Nanodrop 1000(Thermo).

### Stability of dsRNA and confirmation of ingestion of bacteria in the midgut

To confirm that the bacteria expressing dsRNA was introduced into the gut through ingestion, the bacteria were colored in 0.1% FD&C blue solution. After being fed with FD&C colored bacteria for 3 days, midgut was dissected and photographed. To ensure the integrity of dsRNA during feeding period, we did done a test to guarantee the validity of the experiment. The dsRNA on the diet was extracted every one hour and examined on a 2% agarose TBE gel.

### Feeding bioassays

Newly emerged flies were collected on day 0 (within 12 hr after eclosion) and moved into a 30 cm×30 cm×30 cm box. For each treatment 60 flies (sex ratio 1∶1) was reared in each box and the experiment was triplicated. Artificial diet was cut into round piece with diameter 3.2 cm. Each piece was covered by 200 µl dsRNA solution (2 µg/µl) or 200 µl 250× bacteria expressing dsRNA. The artificial diet with dsRNA or bacteria was replaced by new one twice a day. For the bioassay of *rpl19* and *v-ATPase-D*, the adult flies were fed by the dsRNA diet for continuous 14 days. For *noa*, only male flies were fed by the dsRNA diet for 14 days. Then the RNAi individuals were allowed to mate with the virgin control females. For *rab11*, both male and female flies were fed by the dsRNA diet for 14 days. Then the RNAi males were mated with RNAi females. The egg production was counted for continuous 7 days after mating for *noa* and *rab11* group. 6 flies (sex ratio 1∶1) were collected on day 0, 1, 2, 4 and day 7 for qRT-PCR and frozen in liquid nitrogen and kept at −80°C before use. RNAs were extracted from abdomens of flies using RNAiso™ Plus (TaKaRa). Mortality of each group was recorded.

### Confirmation of gene expression in specific tissues

Feeding bioassays were redone as described before to confirm the gene knockdown in specific tissues. In each group, either dsRNA or bacteria expressing dsRNA were added to the artificial diet for continuous 7 days. Samples were collected on different days in each group according to the results in previous bioassays. 2 days with significant changes of gene expression in previous bioassays were chosen to collect samples for each group as follows: both ds-*noa* and ds-*noa* bacterial treatment group on day 2 and day 7; both ds-*rab* and ds-*rab* bacterial treatment group on day 4 and day 7; ds-*v-ATPase-D* treatment group on day 4 and day 7, ds-*v-ATPase-D* bacterial treatement group on day 1 and day 4; ds-*rpl19* treatment group on day 4 and day 7,and ds-*rpl19* bacteira treatment group on day 2 and day 7. Different tissues were isolated from the samples collected in the experiments. RNAs were extracted using RNAiso™ Plus (TaKaRa).

### Real-time PCR

Real-time RT-PCR was performed using One Step SYBR® PrimeScript™ RT-PCR Kit (TaKaRa) according to the manufacturer's instructions on a BioRad iCycler. The assays were triplicated. In order to avoid the disturbance of off target effect, primers for qPCR were designed to detect the part outside the dsRNA fragment. 16S rRNA was chosen as internal control gene after validation. The relative gene expression data were analyzed using 2^−ΔΔ*C*^_T_ method as described by Livak and Schmittgen [45]. The results were analyzed by one-way analysis of variance (ANOVA).

### Statistical Analysis

All the results from experimental replicates were analyzed by one-way analysis of variance (ANOVA) and Duncan's test using SPSS 16.0 (IBM Corporation, Somers, NY). * stands for p-values<0.05 were considered significant.

## References

[1] Stephens AEA, Kriticos DJ, Leriche A (2007). The current and future potential geographical distribution of the oriental fruit fly, *Bactrocera dorsalis* (Diptera: Tephritidae).. Bulletin of Entomological Research.

[2] Fire A, Xu SQ, Montgomery MK, Kostas SA, Driver SE (1998). Potent and specific genetic interference by double-stranded RNA in *Caenorhabditis elegans*.. Nature.

[3] Timmons L, Fire A (1998). Specific interference by ingested dsRNA.. Nature.

[4] Timmons L, Court DL, Fire A (2000). Ingestion of bacterially expressed dsRNAs can produce specific and potent genetic interference in *Caenorhabditis elegans*.. Gene.

[5] Tabara H, Grishok A, Mello CC (1998). RNAi in *C. elegans*: Soaking in the genome sequence.. Science.

[6] Geley S, Muller C (2004). RNAi: ancient mechanism with a promising future.. Experimental Gerontology.

[7] Hannon GJ (2002). RNA interference.. Nature.

[8] Boissona B, Jacquesb JC, Choumetc V, Martina M, Xu JN (2006). Gene silencing in mosquito salivary glands by RNAi.. FEBS Letters.

[9] Ghanim M, Kontsedalov S, Czosnek H (2007). Tissue-specific gene silencing by RNA interference in the whitefly *Bemisia tabaci* (Gennadius).. Insect Biochemistry and Molecular Biology.

[10] Possamai SJ, Trionnaire GL, Bonhomme J, Christophides GK, Rispe C (2007). Gene knockdown by RNAi in the pea aphid *Acyrthosiphon pisum*.. BMC Biotechnology.

[11] Baum JA, Bogaert T, Clinton W, Clinton W, Heck GR (2007). Control of coleopteran insect pests through RNA interference.. Nature Biotechnology.

[12] Turner CT, Davy MW, MacDiarmid RM, Plummer KM, Birch NP (2006). RNA interference in the light brown apple moth, *Epiphyas postvittana* (Walker) induced by double-stranded RNA feeding.. Insect Molecular Biology.

[13] Zhou XG, Wheeler MM, Oi FM, Scharf ME (2008). RNA interference in the termite *Reticulitermes flavipes* through ingestion of double-stranded RNA.. Insect Biochemistry and Molecular Biology.

[14] Bautista MAM, Miyata T, Miura K, Tanaka T (2009). RNA interference-mediated knockdown of a cytochrome P450, CYP6BG1, from the diamondback moth, *Plutella xylostella*, reduces larva resistance to permethrin.. Insect Biochemistry and Molecular Biology.

[15] Araujo RN, Santos A, Pinto FS, Gontijo NF, Lehane MJ (2006). RNA interference of the salivary gland nitrophorin 2 in the triatomine bug *Rhodnius prolixus* (Hemiptera: Reduviidae) by dsRNA ingestion or injection.. Insect Biochemistry and Molecular Biology.

[16] Griebler M, Westerlund SA, Hoffmann KH, Meyerring-Vos M (2008). RNA interference with the allatoregulating neuropeptide genes from the fall armyworm *Spodoptera frugiperda* and its effects on the JH titer in the hemolymph.. Journal of Insect Physiology.

[17] Tian H, Peng H, Yao Q, Chen H, Xie Q (2009). Developmental control of a Lepidopteran pest *Spodoptera exigua* by ingestion of bacteria expressing dsRNA of a Non-Midgut Gene.. PLoS ONE.

[18] Newmark PA, Reddien PW, Cebria F, Alvarado AS (2003). Ingestion of bacterially expressed double-stranded RNA inhibits gene expression in planarians.. Proc Natl Acad Sci USA.

[19] Solis CF, Santi-Rocca J, Perdomo D, Weber C, Guillén N (2009). Use of bacterially expressed dsRNA to downregulate *Entamoeba histolytica* gene expression.. PLoS ONE.

[20] Gura T (2000). A silence that speaks volumes.. Nature.

[21] Daniel RGP, John AG (2008). RNAi-mediated crop protection against insects.. Trends in Biotechnology.

[22] Hannon GJ (2003). RNAi: A Guide to Gene Silencing.

[23] Chen SL, Dai SM, Liu Kh, Chang C (2008). Female-specific doublesex dsRNA interrupts yolk protein gene expression and reproductive ability in oriental fruit fly, *Bactrocera dorsalis* (Hendel).. Insect Biochemistry and Molecular Biology.

[24] Winston WM, Molodowitch C, Hunter CP (2002). Systemic RNAi in *C. elegans* requires the putative transmembrane protein *sid-1*.. Science.

[25] Saleh MC (2006). The endocytic pathway mediates cell entry of dsRNA to induce RNAi silencing.. Nature Cell Biology.

[26] Feo S, Davies B, Fried M (1992). The mapping of seven intron-containing ribosomal protein genes shows they are unlinked in the human genome.. Genomics.

[27] Kitagawa N, Mazon H, Heck AJR, Wilkens S (2007). Stoichiometry of the peripheral stalk subunits E and G of yeast V-ATPase determined by mass spectometry.. The Journal of Biological Chemistry.

[28] Jung A, Hollmann M, Schafer MA (2007). The fatty acid elongase *noa* is necessary for viability and has a somatic role in *Drosophila* sperm development.. Journal of Cell Science.

[29] Giansanti MG, Belloni G, Gatti M (2007). *rab11* is required for membrane trafficking and actomyosin ring constriction in meiotic cytokinesis of *Drosophila* males.. Molecular Biology of the Cell.

[30] Tiwari AK, Roy JK (2008). *rab11* is essential for fertility in *Drosophila* cell biology international..

[31] Bin Z, Liu YH, Zhao LL, Xu Z (2008). Research progress of oriental fruit fly *Bactrocera dorsalis* (Hendel).. Chinese Agricultural Science Bulletin.

[32] Bustin SA, Benes V, Garson JA, Hellemans J, Huggett (2009). The MIQE guidelines-minimum information for publication of quantitative real-time PCR experiments.. Clinical Chemistry.

[33] Saleh MC, Tassetto M, Goic B, Gausson V, Berry B (2009). Antiviral immunity in *Drosophila* requires systemic RNA interference spread.. Nature.

[34] Haque SJ, Williams BR (1998). Signal transduction in the interferon system.. Seminars in Oncology.

[35] Sledz CA, Holko M, de Veer MJ, Silverman RH, Williams BRG (2003). Activation of the interferon system by short-interfering RNAs.. Nature Cell Biology.

[36] Scacheri PC, Rozenblatt-Rosen O, Caplen NJ, Wolfsberg TG, Umayam L (2004). Short interfering RNAs can induce unexpected and divergent changes in the levels of untargeted proteins in mammalian cells.. Proc Natl Acad Sci USA.

[37] Zambon RA, Nandakumar M, Vakharia VN, Wu LP (2005). The Toll pathway is important for an antiviral response in *Drosophila*.. Proc Natl Acad Sci USA.

[38] Dostert C, Jouanguy E, Irving P, Troxler L, Hetru C (2005). The Jak-STAT signaling pathway is required but not sufficient for the antiviral response of *Drosophila*.. Nature Immunology.

[39] Zheng ZM, Tang S, Tao MF (2005). Development of resistance to RNAi in mammalian cells.. Annals of the New York Academy of Sciences.

[40] Gitlin L, Karelsky S, Andino R (2002). Short interfering RNA confers intracellular antiviral immunity in human cells.. Nature.

[41] Jacque JM, Triques K, Stevenson M (2002). Modulation of HIV-1 replication by RNA interference.. Nature.

[42] Bucher E, Hemmes H, de Haan P, Goldbach R, Prins M (2004). The influenza A virus NS1 protein binds small interfering RNAs and suppresses RNA silencing in plants.. J Gen Virol.

[43] Li WX, Li H, Lu R, Li F, Dus M (2004). Interferon antagonist proteins of influenza and vaccinia viruses are suppressors of RNA silencing.. Proceedings of the National Academy of Sciences of the United States of America.

[44] Chang J, Provost P, Taylor JM (2003). Resistance of human hepatitis delta virus RNAs to dicer activity.. Journal of Virology.

[45] Livak KJ, Schmittgen TD (2001). Analysis of relative gene expression data using real-time quantitative PCR and the 2^−ΔΔ*C*^
_T_ method.. Methods.

